# Factors associated with emotional and behavioural problems among school age children of breast cancer patients

**DOI:** 10.1038/sj.bjc.6602887

**Published:** 2005-11-29

**Authors:** M Watson, I St. James-Roberts, S Ashley, C Tilney, B Brougham, L Edwards, C Baldus, G Romer

**Affiliations:** 1 Department of Psychological Medicine, The Royal Marsden Hospital NHS Trust, Downs Road, Sutton, Surrey SM2 5PT, UK; 2 Thomas Coram Research Unit, Institute of Education, University of London, UK; 3 Department of Child and Adolescent Psychiatry & Psychotherapy, University of Hamburg, Germany

**Keywords:** breast cancer, emotional problems, behavioural problems

## Abstract

To identify factors linked with emotional and behavioural problems in school age (6- to 17-year-old) children of women with breast cancer. Reports of children's emotional and behavioural problems were obtained from patient mothers, their healthy partners, the children's teacher and adolescents using the Child Behaviour Checklist and Mental Health subscale of the Child Health Questionnaire. Parents reported on their own level of depression and, for patients only, their quality of life. Family functioning was assessed using the Family Assessment Device and Cohesion subscale of the Family Environment Scale. Using a cross-sectional within groups design, assessments were obtained (*N*=107 families) where the patients were 3–36 months postdiagnosis. Risk of problems in children were linked with low levels of family cohesion, low affective responsiveness and parental over-involvement as reported by both child and mother. Adolescents reported family communication issues, which were associated with externalising behaviour problems. Maternal depression was related to child internalising problems, particularly in girls. Whether the mother was currently on or off chemotherapy was not associated with child problems nor was time since cancer diagnosis. These findings held across child age. Where mothers have early stage breast cancer, a substantial minority of their school-aged children have emotional and behavioural problems. Such cases are characterised by the existence of maternal depression and poor family communication, rather than by the mother's treatment status or time since diagnosis. Targeted treatments, which focus on maternal depression and family communication may benefit the children and, through improved relationships, enhance the patients' quality of life.

Although the emotional impact of cancer on families is clearly documented in relation to partners (Zahlis and Shands, 1991; Glasdam *et al*, 1996; Baider *et al*, 1998; Northouse *et al*, 1998) and adult children (Rait and Lederberg, 1989; Edwards and Clarke, 2004), the evidence for school age children is less clear (Romer *et al*, 2002). Several studies have reported that children of mothers with breast cancer exhibit a high rate of psychological and behavioural disturbance (Wellisch *et al*, 1992; Compas *et al*, 1994; Welch *et al*, 1996; Birenbaum *et al*, 1999; Nelson and While, 2002). However, Hoke (2001) found that children of breast cancer patients diagnosed during the previous year were functioning better than a sample unaffected by cancer. Armsden and Lewis (1994) found that the young (6–12 years) children of nonmetastatic breast cancer patients had significantly lower self-esteem than mothers with benign breast disease and yet had better than average behavioural adjustment than the noncancer group.

One explanation for these apparent inconsistencies is that they are due to variations between studies in the groups included and in how child problems are measured, together with difficulties in obtaining adequate, homogeneous, sample sizes. Arguably, some parents will be less incapacitated by their illness and avoid any impact on their children, while others will manage less well. It follows that rather than approaching parents with cancer as a homogeneous group, there is a need to examine variability *between* subgroups of families, in order to throw light onto the factors which predict where children fare badly. One potential source of variability between cases is in the nature and severity of the illness and the associated treatment. For example, the use of chemotherapy as a treatment for early breast cancer has expanded over the last decade. While this has made a positive impact on patient survival it may have been at a cost to families. The period when women receive chemotherapy may be a time of particular pressure. The frequent hospital visits, physical side-effects of treatment (including alopecia, nausea and emesis, and fatigue), loss of family income through mother's absence from work, and disruption to family routines are likely to increase family stress. As well as variations in patient disease and treatment, families are also likely to differ in their social and psychological resources and supports. Lewis's (2004) review of the evidence concludes that ‘Families do not typically know, understand, interact with, or support children coping with the mother's breast cancer’ (page 290). There is evidence that ill parents may under-estimate or not be aware of difficulties their children have in coping with parental cancer (Nelson *et al*, 1994; Welch *et al*, 1996). Nelson and While (2002) point to the impact of inadequate parental coping as a factor increasing risk in the children. Both low self-esteem and poor adjustment in the parent with cancer had an adverse impact on adjustment in the children across a broad age range (8–16 years). Lewis has emphasised the significant adverse impact of both maternal depression and parenting quality on family and child functioning in cancer affected families (Lewis and Hammond, 1996). It follows that the studies of child disturbances need to assess variations between families in how parents communicate with and support their children.

A third source of variability between studies, and families, is in child characteristics, including age and gender. Previous research suggests that adolescent children experience most problems (Welch *et al*, 1996; Birenbaum *et al*, 1999), with teenage daughters being most at risk (Wellisch *et al*, 1992; Compas *et al*, 1994). Visser *et al* (2005)reported that adolescent daughters were more affected by parent's cancer treatment intensity whereas for boys it was relapse of cancer. However, the evidence on gender differences remains insubstantial given the low number of studies including boys.

The UK NICE Guidelines on Cancer Services; Improving Supportive and Palliative Care for Adults with Cancer (2004), recommend that psychological care is available to families. However, to date there has been almost no development of family- or child-focused psychological services (children with cancer excepted) within cancer centres. By identifying which factors in families result in child disturbances, studies can help to target clinical services and, by supporting families, which are most vulnerable to these problems, improve the quality of life for patients themselves.

Using a relatively large homogeneous sample and standard, well-validated measures, this study aims to clarify the family characteristics, which might contribute to poor adjustment in children. Based on the McMaster model of family functioning and Kissane *et al* (1996) work applying it to cancer, and the cited literature, the main hypotheses tested in the current study are that rates of child emotional and behavioural problems will be higher in families:

- with restricted communication
- with low cohesiveness
- where the patient is currently receiving chemotherapy
- where parents show symptoms of depression.

Lastly, given the still insubstantial evidence on age and gender, the null hypothesis is tested that types and rates of child problems will be similar across age-groups and gender.

## METHOD

### Participants

A consecutive series of early stage breast cancer patients attending outpatient clinics at a large cancer treatment centre (Royal Marsden Hospital, UK) was approached. Eligible families were; patients diagnosed between 3 and 36 months previously, aged between 24 and 55 years (inclusive), having children aged 6–17 (inclusive) with whom they had regular contact sufficient to fulfil a main carer role (defined as physical or telephone contact at least once a week). One target child was selected at random (child whose birthday fell closest to the date of recruitment) from each family for assessment. Where target children were aged 11–17 (inclusive) they were invited to complete a questionnaire. Teachers were asked to provide an assessment of child emotional and behavioural problems at school if the parent with cancer consented. Teachers chosen were those who, in the opinion of the head teacher, worked most with the child over the past 6 months.

### Procedure

Patients were recruited where permission to approach had been agreed by their hospital consultant. The study was approved by the Local Research Ethics Committee and all participants gave written informed consent.

Participants completed the study questionnaires independently of other family members. All adolescents approached were aware of their mother's diagnosis. For younger children, the patients reported that in 76% (32/42) of cases the child had been told of the diagnosis. A cross-sectional within-groups design was used with assessment on one occasion only.

### Measures

The selection of measures was made within the context of a European Union collaborative research group (Children of Somatically Ill Parents Studies – COSIP Protocol Number QLRT-2000-02378) with a number of shared common measures (multinational data to be reported elsewhere).

### Child emotional and behavioural problems

Two measures were used to assess child psychological problems: the *Child Behaviour Checklist* (*CBCL*) which covers parental reports on children ages 6–17 years (Achenbach and Rescorla, 2001) and the Mental Health subscale of the *Child Health Questionnaire* (*CHQ*–*MH*) (Landgraf *et al*, 1999).

For the CBCL, the Youth Self Report Form for 11- to 17-year-old adolescents (YSR) or Teacher Report Form (TRF) versions were completed, as appropriate, in addition to the parental CBCL report form completed by both parents (as available) to assess children's behavioural and emotional functioning. The YSR and TRF are parallel versions of the original CBCL instrument designed to measure the same variables by each type of informant; for brevity's sake they will all be referred to here as ‘CBCL measures’. Following previous studies (Visser *et al*, 2005; Lewis *et al*, 2005), Internalising, Externalising and Total Problem scores will be examined here as indices of child problems. For ethical reasons, six items about bowel and sexual problems were omitted from the CBCL. Although this could lower the Total Problem score, these items are unlikely to apply to children in this study, so that any effects will be minimal. The CBCL is widely used and has good psychometric properties and prior application in an oncology setting (Carpentieri *et al*, 1993; Dolgin *et al*, 1997; Noll *et al*, 1997).

Parents and adolescents also completed the Mental Health subscale of the Child Health Questionnaire (CHQ-MH: CF 87 (Child Form) & PF 98 (Parent Form) as a measure of current child distress. No clinical cutoffs are provided in the manual. Good psychometric properties have been reported on US samples with internal consistency of 0.76 (Landgraf *et al*, 1999). No UK reliability or age/gender specific data are available.

### Parental depression

Parental depression was assessed using the Beck Depression Inventory (BDI-II) (Beck *et al*, 1996), used extensively as a screening tool for depression in normal populations and in the oncology setting (Gray, 1987; Kissane *et al*, 1994; Berard *et al*, 1998). Berard *et al* (1998) confirmed that a score of ⩾16 discriminated ‘true’ cases of depression. Caseness strictly was defined here as scores in the moderate or above range (20 or above according to the BDI manual).

The BDI-II has good psychometric properties with internal consistency of 0.93 reported in the manual.

### Family functioning

Two measures of family functioning, the Family Assessment Device (FAD) and Cohesion subscale of the Family Environment Scale (FES) were completed by patient, healthy parent and adolescent (where the latter was selected as target child).

The FAD (Epstein *et al*, 1983) covers seven dimensions: Problem Solving; Communication; Role Allocation; Affective Responsiveness; Affective Involvement; Behaviour Control; a 12-item General Functioning scale (Epstein *et al*, 1983). Based on the McMaster model of family functioning it assesses structural and organisational characteristics that distinguish between healthy and unhealthy families (Epstein *et al*, 1983). Good psychometric properties have been reported in medical cohorts with alpha coefficients ranging from 0.57 to 0.86 (Kabacoff *et al*, 1990) with previous use in an oncology setting (Byles *et al*, 1988). High scores are interpreted as evidence of family dysfunction. The FAD does not contain a measure of family cohesion, an important element of family coping flagged up by Kissane *et al* (1996), and was supplemented by the cohesion subscale of the FES. This measures cohesion on a 10 point scale with 0–3 representing poor, 4–6 moderate and 7–9 good cohesion. There are no psychometric data available where this subscale has been used in isolation.

### Other measures

The SF-8 version of the Medical Outcomes Health Survey (Ware *et al*, 2001) measures physical and emotional quality of life (QoL). No clinical cutoffs are given and convergent correlations ranging from 0.70 to 0.88 have been reported (Ware *et al*, 2001). A score of 50 is taken to be the average score with higher scores indicating frequency or severity of symptoms. Good psychometric properties have been reported with reliability coefficients of 0.70 or greater for each item (Ware *et al*, 2001) and test–retest reliability ranging from 0.61 to 0.70.

### Disease variables

Data on treatment status (on or off chemotherapy) and time since diagnosis were collected.

### Demographic variables

These included; marital status, employment status of both patient and partner, their level of education achieved, ethnic group and socioeconomic status, number of children in the family, birth order of the child selected for assessment, child's own health status (i.e. whether the child had any medical condition requiring treatment on a regular basis), whether patient was a single parent, age of parents, and with whom child lives. Patients were asked about any treatment for emotional problems, such as depression or anxiety, before their cancer diagnosis or any hospital admission for ‘nervous problems’.

### Statistical methods

The data were analysed in three stages. First, descriptive statistics were generated for each of the measures provided by each of the informants (mother, partner, adolescent, and teacher). For the CBCL, raw Internalising, Externalising and Total Problems scores were transformed to standardised T scores using the CBCL data conversion programme (Arnold *et al*, 2000). To identify child problems, the CBCL and CHQ-MH data were dichotomised into ‘cases’ *vs* ‘noncases’. As no British CBCL norms exist, caseness was defined using the original, American, norms and clinical cutoff scores provided by Achenbach and Rescorla (2001). A problem case was defined by a score provided by any one informant (mother, father, adolescent or teacher) that exceeded the American clinical or borderline-clinical CBCL, YSR or TRF cutoff score for such informants. No clinical cutoffs are available for the CHQ-MH CF87 or PF 98 form subscales. Problem cases were defined as those falling into the worst quartile (parental CHQ-MH score <69; adolescent CHQ-MH score <58).

In Stage 2, a univariate logistic regression analysis determined which variables predicted CBCL or CHQ-MH casesness. Owing to the large number of comparisons significance was set at *P*<0.01. The variables included demographics (maternal age, sex of child, single mothers, mother's past treatment for emotional problems), disease and treatment characteristics (time since diagnosis, current treatment status), FAD and FES scores, parental depression and patient QoL (SF8) scores. Scores from each family member were considered as well as the discrepancy score between mother and adolescent.

In stage 3, a multivariate logistic regression analysis was undertaken using the maximum likelihood method and a step-up procedure. Variables were added to the model in order of increasing significance until no longer significant at the 5% level. A family was excluded from the analysis if any variable was missing. To maximise sample size, the multivariate analysis was confined to maternal predictor variables, since these were available for all families. Alpha coefficients for all measures were examined for our cohort and ranged from 0.616 to 0.914.

## RESULTS

### Participants' demographic characteristics

Of 438 approached, 15 refused at this point, and 170/423 (41%) had children in the target age range, agreed to participate and offered gateway consent to approach their partner and the adolescent if the latter had been selected as the target child. Response rates were: 107/170 (63%) breast cancer patients returned the study questionnaires; 56/98 (57%) adolescents; 59/135 (44%) partners; 48/111(43%) teachers. 76/107 (71%) patients were on chemotherapy treatment at the time of assessment. Median time since diagnosis was 11 months (range 3–58). Median age of participants was: 45 (range 29–56) breast cancer patients; 48 (range 31–65) healthy partners; 12 (range 6–17) children; 15 (range 11–17) adolescents only. Gender of child was: male 46, female 64; for adolescents: male 17, female 39. In 15 families, the target child was the only child; 57 children had one sibling, 26 had two, and 10 had three or more siblings; in 30 cases the target child was the eldest. There were 28 single parent families (25 children lived with the biological mother). There were 71/107 (66%) mother patients and 55/59 (93%) partners currently employed. Median age of mother at target child's birth was 33 (range 20–44). Most participants (85%) were in social class I–III, and 34% of mothers and 33% of fathers were educated to at least university degree level. Three patients reported a previous hospital admission for psychological problems; 19 had previous treatment, for psychological problems (antidepressant or anti-anxiety medication). Ethnic groups were: 89 white; one black African; three black Caribbean; one Chinese; four Indian; nine other. There are no differences between returners and nonreturners of questionnaires on measures of age, time since diagnosis, treatment status or whether the child had been told of their parent's diagnosis.

### Child emotional and behavioural problems

The CBCL findings are summarised in Table 1. There were no differences between boys and girls, or between measures of younger (<11 years) *vs* older (11–17 years) children in mean CBCL Externalising, Internalising or Total Problem T scores. In spite of the T score transformation, adolescents self-rated scores still tended to be higher than those provided by other informants (Table 1). For example, adolescents' Total Problem scores were, on average 2.96 points higher than those given by mothers (*n*=53, *P*=0.05) and 5.25 points higher than scores given by fathers (*n*=33, *P*=0.005). Table 1 also shows the number and percentage of children with clinical or borderline-clinical emotional and behavioural problems. In the American standardisation samples, 16% of children in the general community meet this criterion independent of the informant (Achenbach and Rescorla, 2001). Here, adolescents own reports identified 22–32% of them as having clinical or borderline problems, while mothers identified similarly high problem rates in boys and high internalising problem rates, only, in girls. Teachers reported a high problem rate only in girls. Fathers identified far fewer children and adolescents as having problems. Table 2 breaks the problem cases down according to child age, gender and informant. Father reports have been omitted because of the low problem rates in Table 1, and teacher reports omitted because of the small sample size. The small numbers in some of the cells need to be borne in mind. Except for mother-reported externalising problems in girls, the findings show similar rates of problems, in excess of the American standardisation rates, across the age-groups and boys and girls.

The CHQ-MH mean (s.d.) scores given by mothers, fathers and adolescents, respectively, were 77.47 (13.26), 76.88 (11.97) and 68.76 (17.29). For this scale, a higher score is healthy, so here, too, adolescents report more problems than parents (*P*=0.002). Because of how we defined problem cases using this scale, the rates of problems are necessarily the same across informants. Alpha coefficients for the mother, father and child forms were 0.649, 0.698 and 0.683, respectively.

### Measures of family functioning, parental depression, quality of life and breast cancer

Table 3 provides descriptive statistics for the measures of family functioning, parental depression and (for mothers only) the SF8 QoL measures of Physical and Mental health. Cronbach alphas for the FAD ranged from 0.616 to 0.890. Cronbach Aphas for FES Cohesion were: mother 0.712; father 0.753; adolescent 0.769. Cronbach alphas for the BDI were high for both patient and partners (0.896 and 0.892, respectively). On the BDI, 22/107 (21%) of patient mothers and 6/59 (10%) of well fathers/partners had depression scores in the moderate or severe clinical caseness range.

### Parental and family risk factors linked to child psychological problems

FAD and FES discrepancy (between mothers and adolescents) scores were examined but were largely uninformative, so all analyses used individual scores. Table 4 shows variables significantly (*P*<0.01) associated with CBCL Internalising, Externalising and Total Problems, and CHQ-MH problems.

There was substantial agreement between mothers and their adolescent children about which measures of family functioning predicted child Externalising problems. Externalising problems are more likely when; *mother* reports a worse FAD Role Allocation (*P*=0.002), Affective Involvement (*P*=0.001), Behaviour Control (*P*=0.003), and family General Functioning (*P*=0.003), score. Similarly, child Externalising Problems are predicted where an *adolescent* reports worse FAD Communication (*P*=0.001), Affective Responsiveness (*P*=0.007), Affective Involvement (*P*=0.002), Behaviour Control (*P*=0.004), and family General Functioning (*P*=0.002). Increased reports of Externalising problems are also associated with mother (*P*=0.001) and adolescent (*P*=0.001) reporting lower Cohesion on the FES. Mothers' lower FES Cohesion score also predicted Internalising (*P*=0.01) and Externalising (*P*=0.001) problems and was marginally significant for Total Problems (*P*=0.02). There were also differences between mothers and adolescents about which family variables were predictive. For mothers, measures of family Role Allocation predicted all three forms of child problems. For adolescents, family Communication was associated with child Externalising and Total Problems. Father reports of family function were poor predictors of child problems.

Mother's depression predicted both Internalising (*P*=0.001) and CBCL Total Problems (*P*=0.003); this was also significant (*P*<0.01) for women scoring in the caseness range of ⩾20. Depression scores for well fathers were not associated with increased reporting of CBCL problems. Maternal depression was the only factor associated with CHQ-MH caseness (*P*<0.001; Table 5). Contrary to predictions, physical health factors involving time since diagnosis and whether mother is on or off chemotherapy did not predict children's emotional and behavioural problems.

### Multivariate analysis of parental and family risk factors linked to child psychological problems

Maternal variables (FAD, FES, BDI, SF8 scores) were included, together with demographic (age and sex of child, single-parent family status) and illness (current chemotherapy, time since diagnosis) variables in a maximum likelihood step-up multivariate logistic regression analysis of child problem caseness. Maternal FAD Affective Involvement (intrusive, over-involvement) is the strongest predictor of CBCL Total Problems (*P*=0.005), while a low maternal SF8 Physical Health score adds to the prediction. Where mothers report normal affective involvement and good physical health, five of 27 children (19%) are cases, compared to eight of 15 children (53%) where mothers' affective involvement was intrusive and SF8 physical health score was poor (Table 5a–d).

Maternal depression is the most important predictor of Internalising Problem caseness (*P*=0.002) and, after adjusting for this, FAD Role Allocation (*P*=0.01) and child gender (*P*=0.04) are independently predictive (*P*=0.01). None of the 13 male children where the mother is not depressed and reports a good role score are cases; this compares to six of eight (75%) cases in families with a female target child and a depressed mother reporting a poor role score. For Externalising problems maternal FES Cohesion score is the most important predictive factor (*P*=0.001), while the maternal FAD Behaviour score adds to this prediction. Where mothers report good family cohesion and behaviour, 15 of 74 children (20%) are cases, compared to six of seven children (86%) where mothers report poor family cohesion and behaviour.

Maternal depression was the strongest predictor of CHQ-MH problems and no other variables were predictors of child caseness (Table 5d). In all, 18 of 54 children (33%) of nondepressed mothers are cases, compared to 13 of 22 children (59%) of depressed mothers.

Direct measures of physical health factors, such as time since diagnosis and whether mother is on or off chemotherapy, did not predict which children were problem cases. However, maternal report of poor SF8 Physical Functioning, a measure of perceived physical quality of life, adds to the prediction of child problem cases. Mothers' depression is not associated with chemotherapy: five of 31 (16%) on chemotherapy were BDI cases, compared to 17 of 76 women (22%) who were not on chemotherapy (*P*=0.60).

## DISCUSSION

The primary aim was to understand variability *between* families coping with early stage breast cancer in the factors that predict child emotional and behavioural problems. It was assumed that occurrence of breast cancer in a mother would act as a stressor, but that child problems would be more a function of how families coped with the stress than the existence of breast cancer itself. The findings support this expectation. Measures of maternal disease (time since diagnosis, whether on or off chemo-therapy) were expected to add to predictiveness on the assumption that women receiving chemotherapy, or whose diagnosis was recent, would be more incapacitated and find it more difficult to cope. This did not prove to be the case, although, since 71% of the women were receiving chemotherapy, the findings may under-represent this variable. Moreover, SF8 scores (i.e. physical symptoms subjectively judged to impede quality of life), are associated with increased child problems, suggesting the need for further studies of these variables.

The data confirm that maternal depression and poor family functioning are important in understanding those cases where children have emotional and behavioural problems. Our confidence in this conclusion is increased by the consistency between adolescents and their mothers about which factors predicted child Externalising problems. For both mothers and adolescents, reports of poor behaviour control, intrusive family affective involvement, and poor general family functioning, predicted child Externalising, behaviour problems. Maternal reports of poor behaviour control, combined with poor family cohesion, also increased the likelihood of child Externalising problems in multivariate analysis. In contrast, there was little agreement between mothers and adolescents about the factors which predicted child Internalising and Child Health Questionnaire Mental Health, that is, emotional problems. Adolescent reports about family relationships did not predict these at all, but they were predicted by mothers' reports of their own depression, both in univariate and multivariate regression analysis. In the multivariate analysis, maternal depression combined with poorly defined family roles to increase the likelihood of Internalising problems, particularly in girls. This finding is in keeping with earlier evidence that maternal depression is associated with emotional problems in girls as reported by parents (Lewis and Darby, 2004). Unlike some previous studies, we did not find an increased risk in adolescents particularly; the findings applied across age-groups. Taken together, the findings suggest some distinctness about the factors associated with children's behavioural, as opposed to emotional, problems – with behaviour problems linked mainly to poor family functioning, while emotional problems, particularly in girls, are linked with maternal depression. This conclusion is tentative, in view of the small numbers in the subgroup analyses, but warrants further study because of its importance for understanding the causes of different types of problems.

In contrast to the agreement between adolescents and mothers about rates of Externalising behaviour problems and the factors, which predicted them, fathers identified much lower rates of problems than mothers, adolescents or teachers, and were poor predictors of problems. Although other explanations are possible, it may be that fathers were less in touch with their children's problems than mothers or may have been more focussed on the mother as the patient.

Three limitations of this study, which do not affect its main findings, but bear on how they are interpreted, need to be taken into account. First, because both child behaviour problems and family characteristics were measured concurrently, it is not known whether or not the family factors caused the child problems. The findings are consistent with such an explanation but, since it is unlikely to be possible to measure family characteristics before the onset of breast cancer, clarification of this issue may have to await randomised control studies, which intervene to improve parent–child communication. Second, this study did not set out to assess whether the prevalence of emotional and behavioural problems is higher among children of breast-cancer patients than in the general population. The lack of British normative data from the Child Behavior Checklist and Child Health Questionnaire-Mental Health scale precluded doing so, but in any case our interest was in variability among cases rather than in the overall prevalence of problems. For future research purposes, it is worth noting that rates of some problems reported by adolescents, mothers and teachers were above the US population norms. It seems reasonable to conclude that a substantial minority of children in the present study had emotional and behavioural problems according to the definition used.

The third, related, limitation concerns our definition of child problem ‘caseness’ and, particularly, the decision to include a child reported to have problems by any respondent (mother, father, teacher or child) within the caseness group. The issue of how to deal with cross-informant variability in scores on measures of family functioning and child problems has been discussed (Sawyer *et al*, 1992; Birenbaum *et al*, 1999; Bagley *et al*, 2001). The method used here has been advocated in other clinical studies (Bird *et al*, 1992; Piacentini *et al*, 1992; Arsenealt *et al*, 2003) and, to some extent is a pragmatic response to the lack of an agreed ‘true’ measure of child problems. As noted above, agreement between adolescents and mothers about the family factors associated with Externalising problems, which are overt, is better than their agreement about the correlates of emotional problems, which are less available to public scrutiny. Rather than assuming that reports from any one informant are superior, it may be more fruitful to regard the reports as measures of different phenomena. To address this will require larger sample sizes, which enable the predictors of adolescent, maternal and teacher reports of problems to be scrutinised separately. This was not possible given the low response rates in fathers (44%) and teachers (43%). Future analyses of cross-national COSIP data will attempt to throw light on this issue. In the meantime, the data reported here point to the need to organise and deliver family- and child-focussed support within oncology.

In practical terms, it might be advisable to begin by collecting information from women attending breast cancer clinics on whether they have school age and/or dependent children. However, the main difficulty to providing care for families lies at present in the dearth of any family or child-focussed psychosocial services within oncology. Even referral on to community family therapy teams is limited by the lack of resources for families where the main issue is that a parent has a serious medical illness. While the UK NICE Guidelines (2004) advocate the availability of psychological support for the families of patients there are no clear guidelines on how services could be developed to help with the unmet needs arising in the children of patients. Our data highlight these unmet needs, which have hitherto gone largely unnoticed.

## Figures and Tables

**Table 1:** CBCL: T scores and rates of child emotional and behavioural problems

**Informant**	**Mother**		**Father**		**Teacher**		**Adolescent**	
**Child gender**	**Boy**	**Girl**	**Boy**	**Girl**	**Boy**	**Girl**	**Boy**	**Girl**
*N*	44	61	24	33	17	31	18	38
Mean (s.d.)	50.91	52.07	49.92	48.70	50.88	49.45	53.44	52.34
Internalising T score	(11.98)	(9.76)	(8.25)	(10.35)	(8.83)	(10.78)	(11.64)	(12.73)
Mean (s.d.)	50.70	48.38	44.42	47.52	47.29	49.97	53.28	51.55
Externalising T score	(11.88)	(9.03)	(12.05)	(8.74)	(7.27)	(9.47)	(12.69)	(10.13)
Mean (s.d.)	49.95	49.21	45.58	47.67	48.65	49.68	53.00	52.45
Total problems T score	(12.43)	(10.05)	(9.18)	(9.87)	(7.75)	(10.51)	(12.86)	(11.38)
No. (%)	12	15	1	5	2	7	4	10
Internalising cases	(27)	(25)	(4)	(15)	(12)	(23)	(22)	(26)
No. (%)	12	6	1	3	1	7	5	9
Externalising cases	(27)	(8)	(4)	(9)	(6)	(23)	(28)	(24)
No. (%)	12	8	1	5	1	7	5	12
Total problem cases	(27)	(13)	(4)	(15)	(6)	(23)	(28)	(32)

**Table 2:** Number and % of CBCL problem cases according to child age group, child gender, and informant

**Child sex** **Child age group** **Informant** ***N***	**Boy** **Adolescent** **Mother** **23**		**Boy** **Adolescent** **Adolescent** **18**		**Girl** **Adolescent** **Mother** **41**		**Girl** **Adolescent** **Adolescent** **38**		**Boy** **Age<11** **Mother** **21**		**Girl** **Age<11** **Mother** **20**	
	**Case**	**%**	**Case**	**%**	**Case**	**%**	**Case**	**%**	**Case**	**%**	**Case**	**%**
Internalising	6	26.1	4	22.2	10	24.4	10	26.3	6	28.6	5	25.0
Externalising	6	26.1	5	27.8	3	7.3	9	23.7	6	28.6	3	15.0
Total problems	7	30.4	5	27.8	6	14.6	12	31.6	5	23.8	2	10.0

**Table 3:** Descriptive statistics for the measures of family functioning and parental depression and quality of life [mean (s.d.) values]

**Informant**	**Mother**	**Father**	**Youth**
N^a^	105–107	57–58	56
FAD Problem Solving	1.97 (0.37)	1.95 (0.35)	2.16 (0.41)
FAD Communication	2.01 (0.39)	2.05 (0.35)	2.28 (0.39)
FAD Role Allocation	2.26 (0.37)	2.19 (0.29)	2.33 (0.44)
FAD Affective Responsiveness	1.89 (0.52)	2.06 (0.46)	2.12 (0.52)
FAD Affective Involvement	1.99 (0.42)	2.03 (0.35)	2.18 (0.42)
FAD Behaviour Control	1.75 (0.33)	1.77 (0.35)	1.97 (0.37)
FAD General Functioning	1.81 (0.43)	1.84 (0.41)	2.00 (0.50)
FES Cohesion	7.28 (1.97)	7.64 (1.84)	6.46 (2.25)
BDI	12.40 (8.45)	8.51 (7.19)	—
SF8 Physical	45.42 (10.22)	—	—
SF8 Mental	45.84 (10.69)	—	—

a Sample sizes: Mother: FAD & SF8=105, FES=106, BDI=107; Father FAD & FES=58, BDI=59; Youth FAD & FES=56. Youths did not complete BDI. Only mother completed SF8 measures.

**Table 4:** Variables associated (univariate regression analysis) with CBCL and CHQ-MH caseness significant at the *P*<0.01 level

**Item**	**Informant**	**Internal problems**	**External problems**	**Total problems**	**CHQ-MH**
*Family Assessment Device Scores*					
Role allocation	Mother	*P*=0.001	*P*=0.002	*P*=0.006	NS
Affective involvement	Mother	NS	*P*=0.000	*P*=0.006	NS
Behaviour control	Mother	NS	*P*=0.003	NS	NS
General function	Mother	NS	*P*=0.003	NS	NS
Communication	Adolescent	NS	*P*=0.001	*P*=0.007	NS
Affective responsiveness	Adolescent	NS	*P*=0.007	NS	NS
Affective involvement	Adolescent	NS	*P*=0.002	NS	NS
Behaviour control	Adolescent	NS	*P*=0.004	NS	NS
General function	Adolescent	NS	*P*=0.002	NS	NS
Affective involvement	Father	*P*=0.007	NS	NS	NS
					
*Family Environment Scale – Cohesion*					
Cohesion	Mother	*P*=0.01	*P*=0.000	NS	NS
Cohesion	Adolescent	NS	*P*=0.001	NS	NS
					
*Beck Depression Inventory*					
Score	Mother	*P*=0.000	NS	*P*=0.003	*P*=0.000
Score	Father	NS	NS	NS	*P*=0.000
Case	Mother	*P*=0.000	NS	*P*=0.009	*P*=0.000
Case	Father	NS	NS	NS	*P*=0.002
					
*SF8*					
Mental	Mother	NS	NS	NS	*P*=0.000

**Table 5:** Results of the multivariate logistic regression analysis for maternal reports: (a) CBCL Internalising, (b) CBCL Externalising, (c) CBCL Total problem, and (d) CHQ-MH Cases According to Dimensions of the FAD, FES Cohesion, BDI and Child gender

		**Relative risk of Internalising case**	**95% CI**	**Significance**
*(a) Internalising cases*				
Maternal depression	Case : Noncase	5.5	1.7–17.4	0.002
FAD Role Allocation	Worst : Best quartile	2.5	1.4–4.3	0.01
Sex of child	Female : Male	2.7	1.0–7.1	0.04

		**Relative risk of Externalising case**	**95% CI**	**Significance**
*(b) Externalising cases*				
FES cohesion				
	Moderate : Good cohesion	2.7	1.3–5.6	0.001
	Poor : Good cohesion	7.1	1.6–31.7	
FAD behaviour control	Worst : Best quartile	2.3	1.0–5.1	0.04

		**Relative risk of Total Problems case**	**95% CI**	**Significance**
*(c) Total problem cases*				
FAD Affective Involvement	Worst : Best quartile	3.2	1.3–7.8	0.005
SF8 Physical	Worst : Best quartile	2.3	1.1–5.1	0.03

		**Relative risk of CHQ-MH case**	**95% CI**	**Significance**
*(d) CHQ-MH Cases*				
Maternal depression	BDI Case : Noncase	5.3	2.0–14.4	0.001
